# Tonic GABAergic inhibition, via GABA_A_ receptors containing αβƐ subunits, regulates excitability of ventral tegmental area dopamine neurons

**DOI:** 10.1111/ejn.15133

**Published:** 2021-02-14

**Authors:** Kyoko Tossell, Rakesh A. Dodhia, Benjamin Galet, Olga Tkachuk, Mark A. Ungless

**Affiliations:** ^1^ MRC London Institute of Medical Sciences (LMS) London UK; ^2^ Institute of Clinical Sciences (ICS) Faculty of Medicine Imperial College London London UK

**Keywords:** addiction, extra‐synaptic, midbrain, synaptic transmission

## Abstract

The activity of midbrain dopamine neurons is strongly regulated by fast synaptic inhibitory γ‐Aminobutyric acid (GABA)ergic inputs. There is growing evidence in other brain regions that low concentrations of ambient GABA can persistently activate certain subtypes of GABA_A_ receptor to generate a tonic current. However, evidence for a tonic GABAergic current in midbrain dopamine neurons is limited. To address this, we conducted whole‐cell recordings from ventral tegmental area (VTA) dopamine neurons in brain slices from mice. We found that application of GABA_A_ receptor antagonists decreased the holding current, indicating the presence of a tonic GABAergic input. Global increases in GABA release, induced by either a nitric oxide donor or inhibition of GABA uptake, further increased this tonic current. Importantly, prolonged inhibition of the firing activity of local GABAergic neurons abolished the tonic current. A combination of pharmacology and immunohistochemistry experiments suggested that, unlike common examples of tonic inhibition, this current may be mediated by a relatively unusual combination of α4βƐ subunits. Lastly, we found that the tonic current reduced excitability in dopamine neurons suggesting a subtractive effect on firing activity.

## Graphical abstract

Midbrain dopamine neuron receives a tonic inhibitory current which is sensitive to GABA_A_ receptor antagonists. We found that modification of GABA release or subunit selective GABA_A_ receptor blockage altered this tonic current. This also changed the excitability of dopamine neuron, indicated that this tonic GABAergic current through rare combination of αβƐ subunits has a subtractive effect on firing activity in midbrain dopamine neuron.

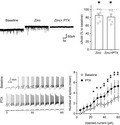

**Abbreviations**
aCSF: artificial cerebrospinal fluid
AMPA: α‐amino‐3‐hydroxy‐5 methyl‐4‐isoxazole‐propionic acid
ANOVA: analysis of variance
BIC: bicuculline
Cm: membrane capacitance
GABA: γ‐Aminobutyric acid
GFP: green fluorescent protein
IgG: immunoglobulin G
IR‐DIC: infra‐red differential interference contrast
mIPSCs: miniature inhibitory postsynaptic currents
NDS: normal donkey serum
NMDA: *N‐methyl‐d‐aspartate*
NO: nitric oxide
PB: phosphate buffer
PBP: parabrachial pigmented area
PBS: phosphate‐buffered saline
PFA: paraformaldehyde
Pitx3: paired‐like homeodomain transcription factor 3
PTX: picrotoxin
SEM: standard error of the mean
sIPSCs: spontaneous inhibitory postsynaptic currents
SNAP: S‐nitroso‐N‐acetylpenicillamine
SNc: substantia nigra pars compacta
TH: tyrosine hydroxylase
THIP: 4,5,6,7‐tetrahydroisoxazolo[5,4‐c]pyridine‐3‐ol
TTX: tetrodotoxin
VTA: ventral tegmental area

## 1 INTRODUCTION

Dopamine neurons in the ventral tegmental area (VTA) and substantia nigra pars compacta (SNc) play key roles in reward processing, appetitive and aversive behaviour, and are implicated in several psychiatric disorders (Fields et al., 2007; Lüscher & Malenka, 2011; Schultz, 2007; Winton‐Brown et al., 2014; Wise, 2004). Dopamine neuron activity is strongly regulated by GABAergic inputs; around half of all synapses in dopamine neurons are GABAergic (Bolam & Smith, 1990; Henny et al., 2012). Dopamine neurons receive GABAergic inputs from VTA GABA neurons, pars reticulate GABA neurons, and GABA neurons in other regions, including the striatum, pallidum, and the rostral medial tegmental nucleus/tail of the VTA (Barrot et al., 2012; Bolam & Smith, 1990; Tepper & Lee, 2007). Recordings from dopamine neurons reveal that they are bombarded with fast inhibitory GABAergic events in vivo and in vitro (Grace & Bunney, 1985; Häusser & Yung, 1994). GABAergic synaptic regulation of dopamine neurons is thought to be important for the generation of bursts (i.e., through disinhibition) and pauses seen in response to salient motivational events in vivo (Brazhnik et al., 2008; Henny et al., 2012; Lobb et al., 2010; Paladini & Roeper, 2014; Paladini & Tepper, 1999; Tepper & Lee, 2007) and the consequent effects on appetitive and aversive behaviour (Parker et al., 2011; Tan et al., 2012; van Zessen et al., 2012). In addition, addictive drugs, including morphine, cocaine and alcohol, can induce long‐term modifications of these synapses that may play a role in the development of addictive behaviour (Bonci & Williams, 1997; Liu et al., 2005; Matsui et al., 2014; Melis & Argiolas, 2002; Nugent et al., 2007; Wanat et al., 2009).

There is growing evidence in other brain regions that low concentrations of ambient GABA can also persistently activate certain subtypes of GABA_A_ receptor, located extrasynaptically, to generate a ‘tonic’ current (Brickley & Mody, 2012; Farrant & Nusser, 2005; Semyanov et al., 2004). Tonic activation of GABA_A_ receptors can increase input conductance which is commonly hyperpolarizing and can lead to shunting of excitatory inputs, therefore playing an important role in regulating neuronal activity. Extrasynaptic GABA_A_ receptors often differ from their synaptic counterparts by exhibiting activation to low concentrations of GABA as well as markedly slower rates of desensitization (Brickley et al., 1996; Bright et al., 2011). The properties of extrasynaptic GABA_A_ receptors are conferred by receptor subunit composition, most commonly the inclusion of α4, α5 or α6, with β and δ subunits (Brickley et al., 2001; Caraiscos et al., 2004; Chandra et al., 2006). Furthermore, the properties of extrasynaptic GABA_A_ receptors are often conferred by their subunit composition differing from their synaptic counterparts. For example, the inclusion of the δ subunit is commonly seen in extrasynaptic receptors, while postsynaptic GABA_A_ receptors typically contain γ subunits (Essrich et al., 1998). Indeed, VTA GABA neurons exhibit a tonic current mediated by δ subunit containing receptors (Vashchinkina et al., 2012, 2014). Of the remaining possible GABA_A_ receptor subunits (i.e. ε, θ, π and ρ1‐3), only Ɛ (Moragues et al., 2000, 2002; Sinkkonen et al., 2000) and θ (Bonnert et al., 1999; Moragues et al., 2002; Sinkkonen et al., 2000) are expressed in the midbrain. Θ subunit is highly selective with subunit pairing to make functional GABA_A_ receptors, and it can recruit γ subunits or Ɛ subunits (Bonnert et al., 1999; Ranna et al., 2006). In contrast, Ɛ subunits can form functional GABA_A_ receptors with αβ subunits which generate tonic GABAergic current (Neelands et al., 1999). Although exogenous application of GABA can induce a tonic current in VTA dopamine neurons (Vashchinkina et al., 2014), it is not clear if dopamine neurons exhibit an endogenous extrasynaptic tonic GABAergic current. We, therefore, sought to examine this by conducting whole‐cell recording from VTA dopamine neurons in acute brain slices from mice, along with immunohistochemical analysis of GABA_A_ receptor subunits.

## 2 MATERIALS AND METHODS

### 2.1 Animal maintenance and breeding

Male Pitx3‐GFP [B6.129P2‐*Pitx3^tm1MLi^*/Mmjax, MMRRC: 41479] (Zhao et al., 2004), TH‐GFP [Tg(Th‐EGFP)21‐31 Koba, MGI: 3774290] (Matsushita et al., 2002; Sawamoto et al., 2001) heterozygous mice and C57BL/6J mice were used for experiments. All breeding and experimental procedures were conducted in accordance with the Animals (Scientific Procedures) Act of 1986 (UK) and approved by Imperial College London's Animal Welfare and Ethical Review Body. All mice were maintained in social groups of 2–4, where possible, with appropriate environmental enrichment (e.g., bedding and tunnels). Mice were kept in 12 hr light/dark cycle with a free access to standard chow and water ad libitum.

### 2.2 Slice preparation

Mice (6–14 weeks) were anaesthetized with isoflurane and decapitated. The brain was rapidly removed and bathed with ice‐cold (0–4°C) artificial cerebrospinal fluid (aCSF, containing (in mM): 120 NaCl, 3.5 KCl, 1.25 NaH_2_PO_4_, 25 NaHCO_3_, 10 glucose, 1 MgCl_2_, 2 CaC_l2_) fully equilibrated with carbogen gas (95% oxygen and 5% carbon dioxide). Two or three coronal brain slices (220 μm thickness) encompassing the VTA were obtained using a vibratome (Leica VT1000S; Leica Microsystems, Wetzlar, Germany) and were maintained in a standard custom‐made maintenance chamber (Edwards et al., 1989) gently and continuously aerated with carbogen gas for at least 90 min at room temperature (20–22°C) before use for electrophysiology.

### 2.3 Electrophysiology

Pitx3‐GFP mice were used for all voltage‐clamp recordings, and a mixture of Pitx3‐GFP and C57BL/6J mice were used for current‐clamp recordings. Acute brain slices were transferred to a slice recording chamber (Scientifica, UK) and were continuously perfused at a rate of 3–5 ml/min with fully oxygenated aCSF at 34°C. Neurons were visualized using infra‐red differential interference contrast (IR‐DIC) under an upright microscope (Olympus BXWI 51, Japan) equipped with a 40x objective (0.8 numerical aperture), an IR filter, DIC optics and a charge coupled device (CCD) video camera (Watec 902H). Dopamine neurons were identified in Pitx3‐GFP mice by the presence of GFP (viewed under fluorescence illumination (Xcite120 unit; EXFO, UK) coupled to a GFP excitation filter) or in C57BL/6J mice by the presence of an Ih current in neurons located in the lateral parabrachial pigmented area (PBP) of the VTA (Ungless & Grace, 2012).

Whole‐cell patch‐clamp recordings were performed with a Multiclamp 700B amplifier (Molecular Devices, CA) using glass microelectrodes (4–6 MΩ) filled with an internal solution containing (in mM): 135 CsCl, 8 NaCl, 10 Cs‐HEPES, 2 EGTA, 0.2 MgCl_2_, 2 NaATP, and 0.3 NaGTP, pH 7.3 (280/285 mOsm). Current clamp recordings were obtained with a pipette solution that contained potassium gluconate instead of CsCl.

For voltage‐clamp recording, neurons were clamped at −70 mV. α‐amino‐3‐hydroxy‐5 methyl‐4‐isoxazole‐propionic acid (AMPA), *N*‐methyl‐d‐aspartate (NMDA), and GABA_B_ receptors were routinely blocked using NBQX (25 μM), D‐AP5 (50 μM), and CGP‐52432 (5 μM), respectively. Miniature inhibitory postsynaptic currents (mIPSCs) were recorded in the presence of 1 μM tetrodotoxin (TTX). To determine average holding currents, a continuous current recording was measured for 60 counts/1 s epoch, discarding those coincident with spontaneous IPSCs (sIPSCs) or mIPSC. A drug effect on the holding current was obtained after at least 10 min of drug application. Tonic GABA_A_ currents (*I*_tonic_) were defined as the difference between the average holding current (*I*_hold_) in control (Baseline) versus that obtained during drug application and reported as Δ*I*_hold_. Frequency, amplitude, and decay time constants of mIPSCs were analysed off‐line with the MiniAnalysis (Synaptosoft). Average IPSC waveforms were constructed using at least 100 IPSCs.

For current‐clamp recordings, neurons were recorded in current‐clamp conditions for a minimum of 1–2 min after breaking into the whole‐cell mode before delivering depolarizing pulses. A series of hyperpolarization steps were initially applied to identify VTA dopamine neurons by the presence of an Ih current. For excitability experiments, neurons were clamped at −60 mV and step current injections of increasing amplitude from 0 to 60 pA (5 pA steps, 500 ms duration) were used to evoke action potentials. To examine the input‐output relationship of the neurons, the injected current was plotted against the number of action potentials obtained.

### 2.4 Data acquisition and analysis

Membrane currents were low‐pass filtered at 5 kHz, digitized at 20 kHz, and analysed using pClamp 9.2 software (Molecular Devices). Access and input resistances were monitored throughout the experiments using a 5 mV voltage step. The access resistance was typically <20 MΩ, and results were discarded if it changed by more than 20%. Membrane capacitance (*C*m) was measured under voltage clamp at −50 mV using a hyperpolarizing 10 mV, 250 ms step. *C*m was measured from the change in membrane charge taken from the integrated capacity transients (pClamp, Molecular Devices). All potentials cited here have not been corrected for liquid junction potentials (9.2 mV, pClamp).

### 2.5 Immunohistochemistry

Pitx3‐GFP, TH‐GFP and C57BL/6J mice (8–10 weeks old) were anaesthetized with pentobarbiturate (Euthatal), and transcardially perfused in an antibody‐specific manner (see below for specific protocols). Brains were removed and post‐fixed in the adequate fixative solution. Coronal sections were prepared with a cryostat at a thickness of 40 μm covering Bregma −3.0 to −3.65 mm and were collected as free‐floating. Expression in the hippocampal area was used for the validation of successful immunostaining for each subunit (Drexel et al., 2015; Kralic et al., 2006).

#### 2.5.1 α1/α2/α4 subunits

Mice were perfused via the ascending aorta with ice cold Tris‐HCl buffer (pH7.4) for 2 min, immediately followed by 4% paraformaldehyde (PFA)/Tris buffer for 5 min. The brain was extracted immediately after decapitation and post‐fixed for 1h. After rinsing with Tris buffer, the brain was stored at −80°C after 30% sucrose/Tris buffer cryoprotection until processing them further. Sections were briefly incubated in 0.3% hydrogen peroxide/Tris buffer for 5 min, then blocked in Tris buffer/0.2% Triton X‐100 /20% normal donkey serum (NDS) for 30 min. Sections were then moved and incubated in primary antibody solution (Tris buffer/0.2% Triton X‐100/2% NDS, Table 1) for 24 hr at 4°C. After several washes in Tris buffer, sections were incubated in secondary antibody solution (Tris buffer/0.2% Triton X‐100/2% NDS, Table 2) for 1 hr at room temperature.

**TABLE 1:** Primary antibodies

Antigen	Type	Dilution	Company	Catalog no.	AB registry
TH	CP	1:1000	Abcam	ab76442	AB_1524535
GFP	CP	1:2000	Abcam	ab13970	AB_300798
Gephyrin	MM	1:1500	Synaptic Systems	147 021	AB_2232546
GABA_A_α1	RbP	1:1000	Alomone Labs	AGA‐001	AB_2039862
GABA_A_α2	RbP	1:1000	Synaptic Systems	224 103	AB_2108839
GABA_A_α3	RbP	1:500	Alomone Labs	AGA‐003	AB_2039866
GABA_A_α4	RbP	1:500	Alomone Labs	AGA‐008	AB_10917596
GABA_A_Ɛ	RbP	1:1000	Alomone Labs	AGA‐015	AB_2340939
GABA_A_θ	RbP	1:1000	Alomone Labs	AGA‐018	AB_2340942

Abbreviations: AB, antibody; CP, Chicken polyclonal; GABA, γ‐Aminobutyric acid; GFP, green fluorescent protein; MM, mouse monoclonal; RbP, rabbit polyclonal; TH, tyrosine hydroxylase.

**TABLE 2:** Secondary antibodies

Antigen	Conjugate	Type	Dilution	Company	Catalog no.	AB registry
Rabbit IgG	Cy^TM^3	DP	1:2000	Jackson ImmunoResearch	711‐165‐152	AB_2307443
Mouse IgG	Cy^TM^5	MP	1:2000	Jackson ImmunoResearch	715‐175‐151	AB_2340820
Chicken IgG	Alexa 488	GP	1:2000	Thermo Fisher Scientific	A11039	AB_2534096
Chicken IgG	Alexa 633	GP	1:2000	Thermo Fisher Scientific	A21103	AB_2535756

Abbreviations: AB, antibody; DP, donkey polyclonal; GP, goat polyclonal; IgG, immunoglobulin G; MP, mouse polyclonal.

#### 2.5.2 α3/θ subunits

Mice were perfused and processed in the same manner as for *α1/α2/α4* subunit staining and cryoprotected and stored for the same duration as previously described. Sections were briefly incubated in 0.3% hydrogen peroxide/Tris buffer for 5 min, and blocked in Tris buffer/5% skimmed milk /20% NDS for 30 min. Sections were then moved and incubated in primary antibody solution (Tris buffer/5% skimmed milk/2% NDS, Table 1) for 24 hr at 4°C. After several washes in Tris buffer, sections were incubated in secondary antibody solution (Tris buffer/5% skimmed milk/2% NDS, Table 2) for 1 hr at room temperature.

#### 2.5.3 ε subunit

Mice were perfused with ice cold oxygenated aCSF for 2 min, immediately followed by ice‐cold 4% PFA/Tris‐HCl buffer (pH7.4) for 5 min via the ascending aorta. The brain was extracted immediately after decapitation and blocks of midbrain were isolated and immersed in ice‐cold fixative for 90 min. The block of brain was stored at −80°C after 30% sucrose/Tris buffer cryoprotection until processing them further. Sections were blocked in Tris buffer/0.2% Triton X‐100/20% NDS for 30 min at RT. Sections were then moved and incubated in primary antibody solution (Tris buffer/0.2% Triton X‐100/2% NDS, Table 1) for 48 hr at 4°C. After several washes in Tris buffer, sections were incubated in secondary antibody solution (Tris buffer/0.2% Triton X‐100/2% NDS, Table 2) for 1 hr at room temperature.

### 2.6 Microscopy

Images were captured with a SP5 (Leica, Germany) confocal microscope with 63 × 1.40 oil‐immersion objective (1.4 NA) and the pinhole was set at 1 Airy unit. Acquisition of triple labelling was taken sequentially. All images were analysed and prepared with Fiji software using Ortho‐slice extractor and cell counter. All pixels below the fixed threshold were removed first. All signals in the cytoplasm were omitted after consulting orthogonal views from the counting. For the quantitative analysis, 3–4 images were taken from the lateral PBP area from at least three brain slices spanning the anterior to posterior axis for each brain. The lateral PBP was identified by the position of the medial terminal nucleus (MT), the medial lemniscus (ml) and TH expression in the SNc. Example images were background‐subtracted, and a low pass filer was applied. The images were shown using the maximal intensity Z projection mode (a stack of 11 confocal images spaced at 0.5 µm for × 2.5 digital zoom (× 100 total magnification), and six confocal images spaced at 0.3 µm for × 6 digital zoom (× 240 total magnification)).

### 2.7 Drugs

Bicuculline (0131, Tocris), CGP 52432 (SML‐0593, Sigma), D‐AP5 (0106, Tocris), GABAzine/SR‐95531 (S106, Sigma), gaboxadol (T101, Sigma), NBQX (N171, Sigma), NO‐711 (N142, Sigma), Picrotoxin (P1675, Sigma), SNAP (0598, Tocris), THIP (T101, Sigma), TTX (1069/1, Tocris) and zinc chloride (Z0152, Sigma).

### 2.8 Statistical analyses

Data are presented as means ± *SEM* (standard error of the mean). *n* = sample number, *N* = animal number, Statistical comparisons were made using paired Student's *t* test or two‐way ANOVA (with Šídák' post hoc tests), and performed using Prism (Graphpad).

## 3 RESULTS

### 3.1 VTA dopamine neurons exhibit a tonic GABAergic current

**FIGURE 1:**
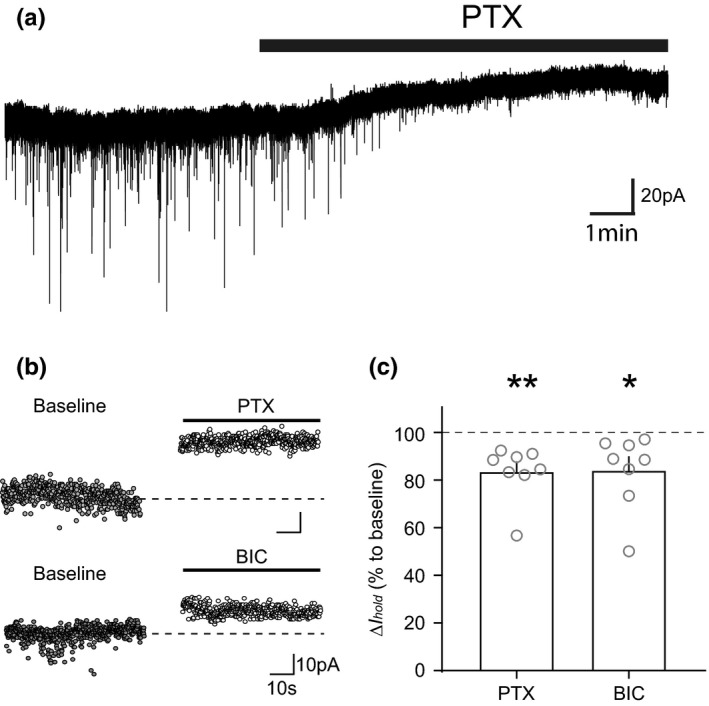
VTA dopamine neurons exhibit a tonic GABAergic current. (a) An example raw trace of holding current change during picrotoxin (PTX, 100 µM) application. (b) An example of average holding current baseline values before and after PTX or bicuculline (BIC, 10 µM) showing a decrease in holding current. (c) Graph showing the mean (+*SEM*) change in holding current following application of PTX and BIC (PTX: *p* = 0.00632, BIC: *p* = 0.0409). Tonic currents were defined as the difference between the average holding current (*I*_hold_) during baseline versus that obtained during drug application as Δ*I*_hold_. *p < 0.05, **p < 0.01

To explore a tonic GABAergic current in neuronal subtype specific manner, we used Pitx3‐GFP mice which selectively express green fluorescent protein (GFP) in VTA dopamine neurons (Zhao et al., 2004). In order to magnify GABA_A_ receptor‐mediated chloride currents, we chose to use the internal pipette solution containing a high concentration of chloride and hold cell membrane at −70 mV to observe inward current. Ionotropic glutamate (AMPA/kainite and NMDA) and GABA_B_ receptors were routinely blocked by adding NBQX (25 µM), D‐AP5 (50 µM) and CGP‐52432 (5 µM), respectively, to the extracellular solution. We first examined whether VTA dopamine neurons expressed a tonic GABAergic conductance by measuring the change in baseline holding current (*I*_hold_) produced by blocking GABA_A_ receptors with the non‐competitive GABA_A_ receptor antagonist, picrotoxin (PTX, 100 µM). We calculated the tonic GABA_A_ receptor‐mediated current (*I*_tonic_) as the change in holding current from baseline (*I*_hold_) after a 5 min application of picrotoxin. In addition to abolishing all spontaneous inhibitory postsynaptic currents (sIPSCs), we found that PTX significantly decreased *I*_hold_ in VTA dopamine neurons (Figure 1a‐c, *t*_7_ = 4.395, *p* = 0.00632, *n* = 8 [*N* = 6]), indicative of a tonic GABAergic conductance. Application of bicuculline (BIC, 10 µM), a competitive GABA_A_ receptor antagonist, also abolished sIPSCs and decreased *I*_hold_ in VTA dopamine neurons (Figure 1b,c; *t*_7_ = 2.502, *p* = 0.0409, *n* = 7 [*N* = 6]). Taken together, these results indicate that VTA dopamine neurons exhibit an endogenous background tonic GABA_A_ receptor‐mediated current.

### 3.2 Tonic GABA_A_ current in VTA dopamine neurons is sensitive to exogenous GABA concentration and maintained by VTA GABA firing neuron activity

**FIGURE 2:**
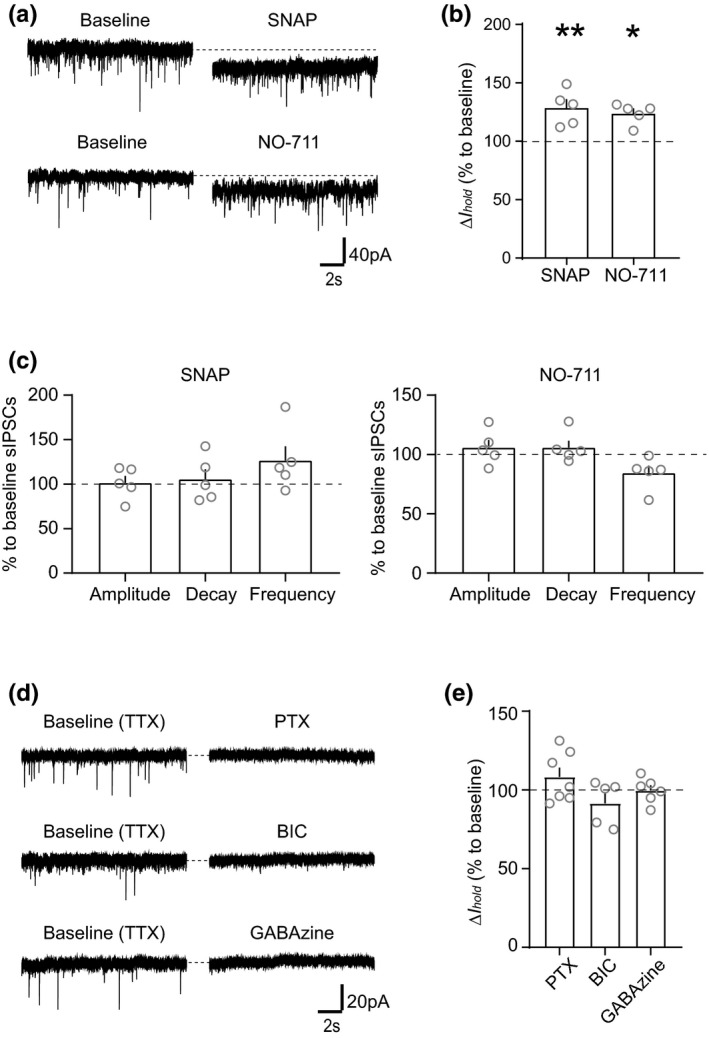
Tonic GABAergic current is sensitive to exogenous GABA concentration and maintained by firing activity of VTA GABA neurons. (a) Example traces and (b) graph of mean (+*SEM*) Δ*I*_hold_ effect showing that bath application of SNAP (400 µM) and NO‐711 (20 µM) increases *I*_hold_ (SNAP: *p* = 0.0073, NO‐711: *p* = 0.013). (c) Graph showing the mean (+*SEM*) effect of SNAP and NO‐711 on sIPSC amplitude, decay time and frequency (SNAP; Amplitude: *p* = 0.8806, Decay time: *p* = 0.8610, Frequency: *p* = 0.1970, NO‐711; Amplitude: *p* = 0.0917, Decay time: *p* = 0.1630, Frequency: *p* = 0.0771). (d) Example traces and (e) graph of mean (+*SEM*) Δ*I*_hold_ effect showing that pro‐longed incubation with TTX (1 µM), to wash‐off exogenous GABA, eliminates the effect of GABA_A_ receptor antagonists, PTX (100 µM), BIC (10 µM) and GABAzine (100 µM) on Δ*I*_hold_ (PTX: *p* = 0.5928, BIC: *p* = 0.9660, GABAzine: *p* = 0.9589). *p < 0.05, **p < 0.01

The presence of a tonic conductance suggests that GABA must be present in the extracellular space at a sufficiently high concentration to cause persistent receptor activation and/or that these receptors open spontaneously. Identifying the source, or sources, of GABA is important for understanding how this tonic receptor activation is modulated. In some neuronal populations, such as in cerebellar granule cells, action potential‐dependent vesicular release plays an important role (Brickley et al., 1996; Kaneda et al., 1995; Wall & Usowicz, 1997). However, some non‐vesicular sources of GABA have also been suggested, including astrocytic release (Kozlov et al., 2006; Liu et al., 2000) and reversal of GABA transporters (Gaspary et al., 1998; Richerson and Wu, 2003). To explore this in VTA dopamine neurons, we firstly examined whether globally increasing GABA could affect this tonic current. Nitric oxide has been shown to increase GABA release at several synapses through potentiating presynaptic GABA release in either a Ca^2+^‐dependent manner or by reversing GABA transporters (Li et al., 2002; Yu & Eldred, 2005). Moreover, the global increase in GABA release that occurs with nitric oxide signalling has been shown to selectively enhance the tonic GABA conductance in thalamic neurons (versus manipulations that increase local GABA release; Bright and Brickley, 2008). The nitric oxide donor, S‐nitroso‐N‐acetylpenicillamine (SNAP), has been shown to potentiate IPSCs in dopamine neurons (Nugent et al., 2007) and could therefore be involved in modulation of the tonic current. We found that application of SNAP (400 µM) produced a dramatic increase in *I*_hold_ (Figure 2a,b; *t*_4_ = 5.046, *p* = 0.0073, *n* = 5 [*N* = 4]), while no significant effects were found on sIPSCs (Figure 2c: Amplitude: *t*_5_ = 0.1601, *p* = 0.8806, *n* = 5 [*N* = 4), Decay time: *t*_5_ = 0.1867, *p* = 0.8610, *n* = 5 [*N* = 4), Frequency: *t*_5_ = 1.546, *p* = 0.1970, *n* = 5 [*N* = 4]).

GABA transporters have well‐documented effects on phasic inhibition, but they also have an important and dynamic influence on ambient GABA, and thus the amount of GABA available to activate extrasynaptic GABA_A_ receptors that mediate tonic currents. For example, in dentate gyrus granule cells (Nusser & Mody, 2002) and hippocampal interneurons (Semyanov et al., 2003) the GABA uptake system can have a profound influence on the amount of tonic current. Astrocytic GABA transporters were also reported to have a possible role in regulating the extracellular GABA during excessive GABA release by the elevated network activity (Kersanté et al., 2013). This suggests that in acute brain slice preparations, GABA uptake may keep the ambient GABA concentration sufficiently low to prevent the activation of GABA_A_ receptors or to allow activation of only a specific subset of receptors such as the high affinity, non‐desensitizing receptors often involved in generating tonic currents. It is possible, therefore, that inhibition of GABA uptake could be used to selectively modulate the tonic current in VTA dopamine neurons. To test this, we used the specific GAT‐1 GABA transporter blocker, NO711, to examine changes in *I*_hold_. We found that NO‐711 (20 µM) increased *I*_hold_ (Figure 2a,b; *t*_4_ = 4.268, *p* = 0.013, *n* = 5 [*N* = 4]), indicating that globally enhanced GABA release can increase the tonic current in VTA dopamine neurons. Additionally, like with SNAP application, NO‐711 application did not significantly change sIPSCs properties in recorded neurons (Figure 2c: Amplitude: *t*_5_ = 2.209, *p* = 0.0917, *n* = 5 [*N* = 4), Decay time: *t*_5_ = 1.707, *p* = 0.1630, *n* = 5 [*N* = 4), Frequency: *t*_5_ = 2.366, *p* = 0.0771, *n* = 5 [*N* = 4]), suggesting that these extrasynaptic GABA_A_ receptors are activated separately from postsynaptic receptors.

We next wanted to examine the possibility that spontaneous activity of VTA GABA neurons may be the source of the tonic current in dopamine neurons. Extracellular GABA level decreases by 70% when action potentials were blocked for more than 20min (De Groote & Linthorst, 2007; Rowley et al., 1995). Here, we found that the prolonged incubation (>30 min) with a voltage‐gated sodium channel blocker, tetrodotoxin (TTX, 1 µM), eliminated the PTX‐sensitive tonic current *I*_hold_ (Figure 2d,e; *t*_6_ = 0.5646, *p* = 0.5928, *n* = 7 [*N* = 6]). Similar results were found with either BIC (10 µM) or GABAzine (a selective and allosteric inhibitor of channel opening of the GABA_A_ receptor for GABA‐induced Cl^‐^ currents) (100 µM) application (Figure 2d,e; BIC: *t*_8_ = 0.04398, *p* = 0.966, *n* = 9 [*N* = 6), GABAzine: *t*_5_ = 0.05412, *p* = 0.9589, *n* = 6 [*N* = 5]). These results suggest that the endogenous tonic current is a result of spontaneous firing activity of VTA GABA neurons. Together with results from NO‐711 and SNAP application, it also suggests that tonic current requires a decent amount of GABA spillover, while the GABA transporter in both neurons and astrocytes are actively regulating the tonic current under basal condition.

### 3.3 γ and δ subunit containing GABA_A_ receptors do not contribute to the tonic current observed in VTA dopamine neurons

**FIGURE 3:**
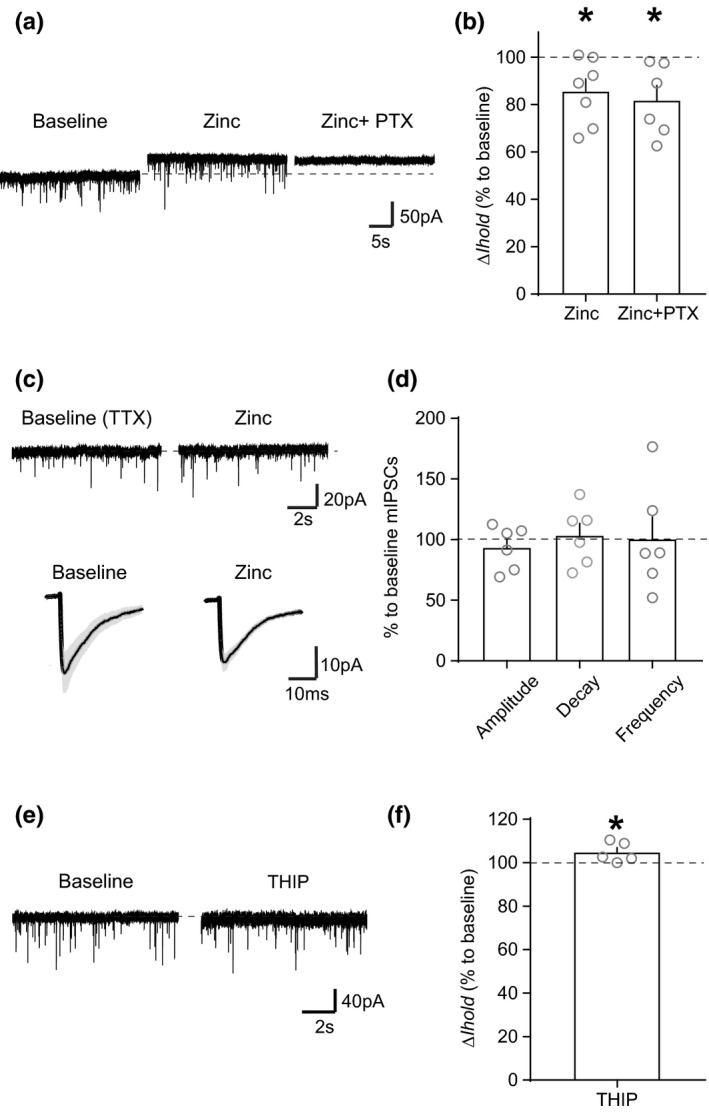
Zinc blocks tonic GABAergic current with little effect on synaptic currents. (a) An example trace showing that bath application of low concentration of Zinc (20 µM) leads to a reduction in *I*_hold_, consistent with the presence of a tonic current. (b) Graph of mean (+*SEM*) Δ*I*_hold_ effect of Zinc and PTX on the holding current (Zinc: *p* = 0.0366, Zinc + PTX: *p* = 0.0492). (c) An example trace and averages (grey trace is *SEM*) of mIPSCs before and after zinc application showing no effect on postsynaptic IPSCs. (d) Graph of mean (+*SEM*) effect of zinc on mIPSC amplitude, decay time and frequency (Amplitude: *p* = 0.2684, Decay time: *p* = 0.8043, Frequency: *p* = 0.8097). (e) An example trace showing that bath application of THIP (5 µM) has only a small effect on *I*_hold_. (f) Graph of mean (+*SEM*) Δ*I*_hold_ effect of THIP on the holding current (*p* = 0.0398). *p < 0.05

GABA_A_ receptors that contain γ subunits are generally found in the postsynaptic GABA_A_ receptor and importantly are relatively insensitive to Zn^2+^ (Essrich et al., 1998; Smart et al., 1991; Stórustovu & Ebert, 2006). We found that Zn^2+^ (20 µM) inhibited the tonic current (Figure 3a,b; Baseline versus Zinc: *t*_6_ = 2.678, *p* = 0.0366, *n* = 7 [*N* = 6), Baseline versus Zinc + PTX: *t*_5_ = 2.585, *p* = 0.0492, *n* = 6 [*N* = 6]). In addition, we observed no significant effects of Zn^2+^ on miniature postsynaptic inhibitory currents (mIPSCs) (Figure 3c,d; Amplitude: *t*_5_ = 1.245, *p* = 0.2684, *n* = 6 [*N* = 6), Decay time: *t*_5_ = 0.2613, *p* = 0.8043, *n* = 6 [*N* = 6), Frequency: *t*_5_ = 0.2538, *p* = 0.8097, *n* = 6 [*N* = 6]). These results suggest that the endogenous tonic current‐mediating GABA_A_ receptors do not contain the γ subunit and are located extrasynaptically, while postsynaptic GABA_A_ receptors, which are responsible for mIPSCs, contain the γ subunit in VTA dopamine neurons.

It has been shown that the δ subunit does not play a role in the tonic current induced by exogenous application of GABA in VTA dopamine neurons (or indeed in synaptic currents in these neurons) but does play a role in VTA GABA neurons (Vashchinkina et al., 2014). Consistent with this, even when we applied a relatively high concentration of THIP (4,5,6,7‐tetrahydroisoxazolo[5,4‐c]pyridine‐3‐ol, 5 µM), a δ‐preferring GABA_A_ receptor agonist, we observed a very small change in *I*_hold_ in VTA dopamine neurons (Figure 3e,f; *t*_4_ = 3.003, *p* = 0.0398, *n* = 5 [*N* = 5]), suggesting that δ subunit containing receptors do not play a role in tonic inhibition in VTA dopamine neurons.

### 3.4 Evidence that Ɛ subunit containing GABA_A_ receptors are located extrasynaptically in VTA dopamine neurons

Electrophysiology results suggest a number of possibilities regarding the subunit composition of the receptors mediating this tonic current in VTA dopamine neurons. One possibility is that they contain only α and β subunits. However, THIP, which had only a small effect on dopamine neurons (Figure 3e,f), has been shown to act as an agonist at αβ receptors (Stórustovu & Ebert, 2006). Moreover, αβ receptors exhibit spontaneous opening, whereas we did not observe a tonic current in the absence of endogenously released GABA (i.e., in the presence of TTX).

**FIGURE 4:**
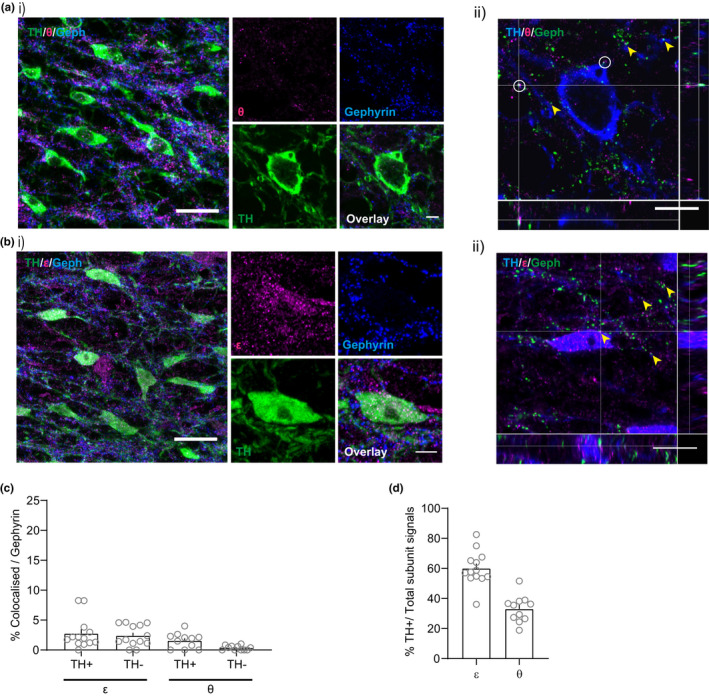
Ɛ subunit containing (but not θ) GABA_A_ receptors are located extrasynaptically in VTA dopamine neurons. (a, i), Representative immunofluorescent images showing that the θ subunit is sparsely expressed in the VTA and rarely colocalized with TH. (ii) Orthogonal view of confocal image showing rare co‐localization of θ and gephyrin (Geph). (b, i) Representative immunofluorescent images showing that the Ɛ subunit is expressed in the VTA in TH+ neurons and shows little co‐localization with gephyrin. (ii) Orthogonal view shows appositive expression of ε and gephyrin. (c) The percentage of colocalized signals against total gephyrin + puncta in both TH+ and TH‐ structures (ε: 2.77% ± 0.73 (TH+), 2.39% ± 0.49 (TH‐), *n* = 13 [*N* = 4), θ: 1.58% ± 0.38 (TH+), 0.35% ± 0.12 (TH‐), *n* = 11 [*N* = 4]). (d) The percentage of subunit expression in the TH+ structure against total subunit expression (ε: 60.02% ± 3.16, *n* = 13 [*N* = 4), θ: 33.07% ± 2.70, *n* = 11 [*N* = 4]). White circles indicate co‐localization of subunits and gephyrin and yellow arrowhead indicate apposition of subunits and gephyrin. Scale bars: (i) 20 µm, inset 5 µm, (ii) 10 µm

Of the remaining possible GABA_A_ receptor subunits that may be present, only ε and θ subunits are found to be expressed in midbrain. Previous reports suggested that both Ɛ subunits and θ subunits are expressed at high levels in the SNc, while not detected in the VTA (Bonnert et al., 1999; Moragues et al., 2000; Okada et al., 2004). However, immunostaining in the VTA can be challenging, and we therefore hypothesized that it may be possible to detect Ɛ and/or θ subunits in the VTA using methods optimized for receptor subunit immunostaining (Notter et al., 2014). Using this approach, however, we observed limited θ subunit expression in dopamine neurons in the VTA (Figure 4a(i,ii),d; % TH+/Total subunit signals; 33.07% ± 2.70, *n* = 11 [*N* = 4]). In contrast, we observed that Ɛ subunits expression was colocalized with dopamine neurons in the VTA (Figure 4b,d; TH+/Total subunit signals; 60.02% ± 3.16, *n* = 13 [*N* = 4]). In addition, it was generally not colocalized with gephyrin, a postsynaptic marker of GABAergic synapses (Essrich et al., 1998; Fritschy et al., 2008; Kneussel & Loebrich, 2007) (Figure 4c; Co‐localization/Gephyrin (TH+); 2.77% ± 0.73, *n* = 13 [*N* = 4]), suggesting that it may be located extrasynaptically, consistent with a role in tonic inhibition.

### 3.5 α4 subunit containing GABA_A_ receptors are expressed extrasynaptically in VTA dopamine neurons

Interestingly, extrasynaptic GABA_A_ receptors are more likely to contain α subunits, particularly α5. However, in situ hybridization, single‐cell PCR and immunostaining studies do not find that α5 is present in the VTA (Allen Mouse Brain Atlas (AMBA; Lein et al., 2007); Ciccarelli et al., 2012; Fritschy & Mohler, 1995; Guyon et al., 1999; Hörtnagl et al., 2013; Okada et al., 2004; Pirker et al., 2000). We, therefore, wanted to examine the expression of other α subunits in the VTA to identify possible candidate members of extrasynaptic GABA*_A_* receptors in VTA dopamine neurons. Previous reports suggested that α1‐4 are expressed in the VTA while α5 and α6 are consistently absent (Fritschy & Mohler, 1995; Hörtnagl et al., 2013; Okada et al., 2004; Tan et al., 2010). We, therefore, used our optimized immunostaining protocol to examine the expression of α1‐4 and co‐localization with TH, gephyrin (a postsynaptic marker).

**FIGURE 5:**
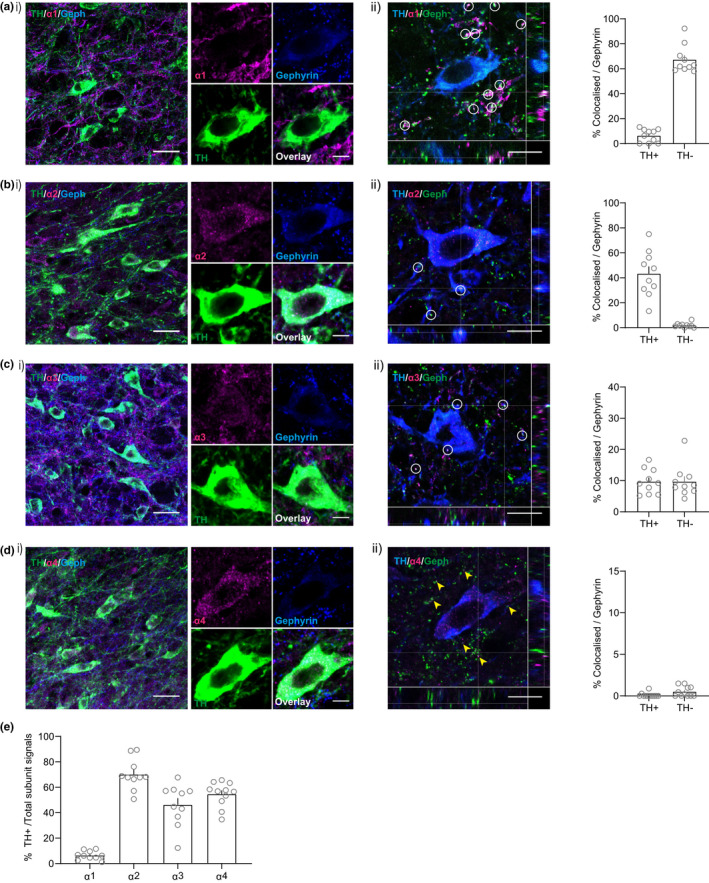
Amongst α1‐4, the α4 subunit is rarely expresses colocalized with postsynaptic markers in VTA dopamine neurons. (a) α1 subunit expresses selectively to non‐TH+ structure. (ii) Co‐localization with gephyrin was found in non‐TH dendritic structures (TH+: 6.45% ± 1.67, TH‐: 67.36% ± 3.53, *n* = 10 [*N* = 4]). (b) α2 subunit expresses strongly on TH+ structures. (ii) Co‐localization with gephyrin was found in TH+ structures (TH+: 43.34% ± 5.79, TH‐: 1.91% ± 0.59, *n* = 10 [*N* = 4]). (c) α3 subunit expresses on both TH+ and TH‐ structures and colocalized with gephyrin (TH+: 9.71% ± 1.26, TH‐: 9.73% ± 1.63, *n* = 10 [*N* = 3]). (d) α4 subunit expresses predominantly to TH+ structures, while these signals rarely colocalized with gephyrin (TH+: 0.11% ± 0.08, TH‐: 0.51% ± 0.19, *n* = 11 [*N* = 5]). (e) The percentage of subunit expression in the TH+ structure against total subunit expression (α1: 6.44% ± 1.10, *n* = 10 [*N* = 4), α2: 70.09% ± 3.85, *n* = 10 [*N* = 4), α3: 46.26% ± 5.18, *n* = 10 [*N* = 3), α4: 54.59% ± 2.93, *n* = 11 [*N* = 5]). White circles indicate co‐localization of subunits and gephyrin and yellow arrowhead indicate apposition of subunits and gephyrin. Scale bars: (i) 20 µm, inset 5 µm, (ii) 10 µm

Previous reports suggest that α1 subunits selectively expressed in VTA GABA neurons (Ciccarelli et al., 2012; Okada et al., 2004; Tan et al., 2010). Consistent with this, we observed very limited α1 subunit co‐localization with TH (Figure 5a(i),e; TH+/Total subunit signals; 6.44 ± 1.10%, *n* = 10 [*N* = 6]). In addition, α1 expression in TH‐ structures was typically colocalized with gephyrin (Figure 5a(ii); Co‐localization/Gephyrin (TH‐); 67.36% ± 3.53, *n* = 10 [*N* = 4]), suggesting that in the VTA α1 subunits are found in postsynaptic GABA_A_ receptors in VTA GABA neurons, but not dopamine neurons.

Evidence for the presence of α2 subunits in VTA is mixed (Ciccarelli et al., 2012; Fritschy & Mohler, 1995; Hörtnagl et al., 2013; Okada et al., 2004; Pirker et al., 2000; Schwarzer et al., 2001), suggesting that it may be expressed at low levels, if at all. Consistent with this, we found that α2 was expressed sparsely, but selectively, in VTA dopamine neurons (Figure 5b(i),e; TH+/Total subunit signals; 70.09 ± 3.85%, *n* = 10 [*N* = 3]). In addition, it was typically colocalized with gephyrin (Figure 5b(ii); Co‐localization/Gephyrin (TH+); 43.34% ± 5.79, *n* = 10 [*N* = 4]), suggesting that α2 subunits are occasionally found in postsynaptic GABA_A_ receptors in VTA dopamine neurons.

Several reports indicate that α3 subunits are reliably expressed in the VTA mostly in dopamine neurons (Okada et al., 2004; Tan et al., 2010). Consistent with this, GABA‐evoked currents in midbrain dopamine neurons in α3 global knockout mice are reduced (Yee et al., 2005). We observed robust α3 subunit expression in both TH+ and TH‐ structures (Figure 5c(i),e; TH+/Total; 46.26 ± 5.18%, *n* = 10 [*N* = 3]), which was rare but colocalized with gephyrin (Figure 5c(ii); Co‐localization/Gephyrin (TH+); 9.71% ± 1.26, *n* = 10 [*N* = 3]), suggesting that α3 subunits are found in synaptic GABA_A_ receptors in VTA dopamine neurons.

Lastly, we examined the expression of α4 subunits which have previously been shown to be expressed in the VTA, including specifically in dopamine neurons (Guyon et al., 1999; Okada et al., 2004). Our immunostaining showed α4 expression selectively in VTA dopamine neurons (Figure 5d(i),e; TH+/Total subunit signals; 54.59 ± 2.93%, *n* = 11 [*N* = 5]). Interestingly, α4 was rarely colocalized with gephyrin (Figure 5d(ii); Co‐localization/Gephyrin (TH+); 0.10% ± 0.08, *n* = 11 [*N* = 5]). These results suggest that α4 subunits is expressed in extrasynaptic GABA_A_ receptors in VTA dopamine neurons.

### 3.6 Tonic GABA currents shunt depolarizing inputs in VTA dopamine neurons to reduce excitability

**FIGURE 6:**
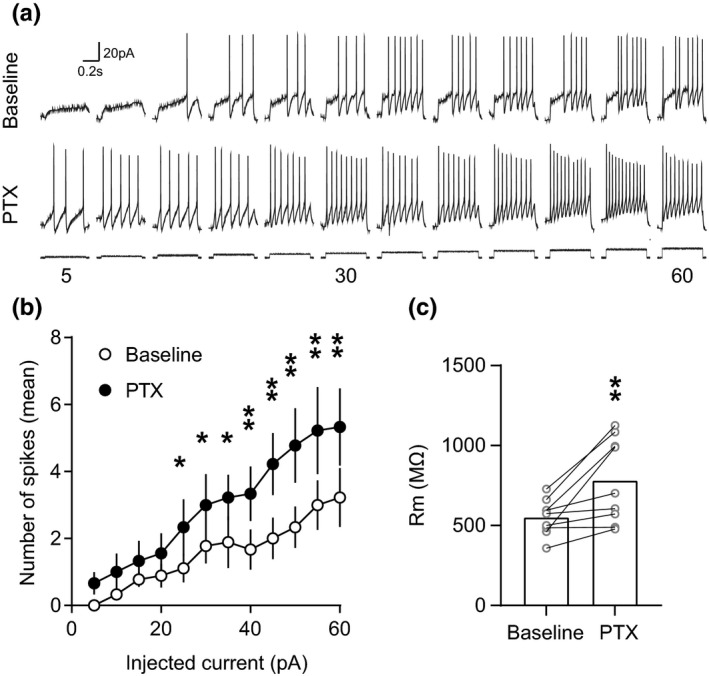
Tonic GABA current reduces excitability in VTA dopamine neurons. (a) Example traces of firing activity in response to current injections of increasing amplitude (starting holding potential of −60 mV) before and during the blockade of the tonic GABAergic current with PTX. (b) Graph of mean (+*SEM*) firing activity showing an increase in excitability following blockade of the tonic current (Group: *p* = 0.0116, Step: *p* = <0.0001, Interaction: *p* = 0.011). (c) Graph of mean (+*SEM*) input resistance (Rm) showing an increase after the blockade of the tonic GABAergic current with PTX (Before‐after interaction: *p* = 0.0093). *p < 0.05, **p < 0.01

Finally, we wanted to examine the functional consequences of this tonic inhibition on dopamine neuron firing activity. Although in other neuronal types the functional consequences of tonic inhibition are not fully understood, one straightforward outcome is a persistent increase in the cell's input conductance. This affects the magnitude and duration of the voltage response to an injected current and increases the decrement of voltage with distance. Consequently, for a given excitatory input, the size and duration of the excitatory postsynaptic potential will be reduced, and the temporal and spatial window over which signal integration can occur will be narrowed, making it less likely that an action potential will be generated. Indeed, several studies have shown that blockage of tonic inhibition with GABA_A_ antagonists decreases the current that is required to achieve a given firing rate (i.e., input‐output curves are shifted; Brickley et al., 1996; Hamann et al., 2002; Semyanov et al., 2003). It is also possible that shunting inhibition can change the slope of the input‐output relationship and hence modulate neuronal gain; that is, it can alter the sensitivity of a neuron to changes in the excitatory input rate (Mitchell & Silver, 2003). Given that the tonic current in VTA dopamine neurons can be decreased by blocking GABA_A_ receptors with PTX, we investigated how this manipulation of the tonic current would influence neuronal excitability. To do this, we used step current injections of increasing amplitude (5–60 pA in 5 pA steps, 500 ms duration, holding potential of −60 mV) to evoke action potentials under baseline conditions and in the presence of PTX in Pitx3‐GFP or C57BL/6J mice (Figure 6a). VTA dopamine neurons were identified using GFP expression in Pitx3‐GFP mice or by the presence of an Ih current in C57BL/6J mice. We found the removal of the tonic current significantly shifted the input‐output relationship to the left (Figure 6b; main effect of group *F*_1,8_ = 10.61, *p* = 0.0116, main effect of current step *F*_11,88_ = 20.9, *p* = <0.0001, interaction: *F*_11,88_ = 3.192, *p* = 0.0011; *n* = 9 [*N* = 9). Importantly, input resistance of recorded neurons was significantly increased after PTX application (Figure 6c; Baseline 551.3 ± 37.22 MΩ, PTX 782.3 ± 88.08 MΩ, *t*_9_ = 3.401, *p* = 0.0093, *n* = 9 [*N* = 9]). PTX is a non‐competitive blocker for GABA_A_ receptor chloride channels and known to also act on GABAc receptors and glycine receptors. However, GABAc receptors are unlikely to be found on VTA dopamine neurons (Petri et al., 2002) and glycine receptors are reported to be insensitive to PTX in adult rats (Ye et al., 1998; Zheng & Johnson, 2001). Thus, the effect of PTX in this study is likely selective to GABA_A_ receptors. This suggests that tonic activation of GABA_A_ receptors can indeed influence the excitability of VTA dopamine neurons, and therefore may represent an important modulator of neuronal activity in vivo.

## 4 DISCUSSION

GABAergic inhibition is essential for regulating the firing pattern of midbrain dopamine neurons and represents a significant proportion of the total synaptic inputs onto these neurons. Although fast phasic synaptic inhibition arising through the activation of postsynaptic GABA_A_ receptors has been well‐described, little is known about whether this strong GABAergic drive can also mediate a tonic conductance. Here, we have shown that tonic GABA_A_ receptor‐mediated currents are also present in VTA dopamine neurons. Our results suggest that the source of this background tonic inhibition is spill over from spontaneously active local GABAergic neurons in the VTA. We also found that the GABA transporter plays an active role in maintaining this background tonic current, and it may be that it can modify its activity to compensate for moderate increases in ambient GABA. However, under conditions where GABA release is significantly increased in a global manner (i.e., when we applied a NO donor), the tonic current can be increased.

Unlike the predominate forms of tonic inhibition in other brain regions, tonic inhibition in VTA dopamine neurons does not appear to involve GABA receptors containing either δ or α5 subunits. Although immunostaining and in situ hybridization studies show that the δ subunit is expressed in the VTA (Hörtnagl et al., 2013; Pirker et al., 2000), single‐cell PCR studies suggest that it is generally absent in VTA/SNc dopamine neurons (Guyon et al., 1999; Okada et al., 2004). In addition, electrophysiological studies show that δ subunit specific agonists enhance tonic currents induced by exogenous GABA in VTA GABA neurons, but not in VTA dopamine neurons (Vashchinkina et al., 2014). Consistent with this, we found that even a relatively high concentration of THIP had a minimal effect on holding currents. Moreover, a δ subunit specific agonist significantly reduces sIPSCs in dopamine neurons (presumably as a consequence of inhibition of VTA GABA neurons; Vashchinkina et al., 2012). With respect to α5 subunits, in situ hybridization, single‐cell PCR and immunostaining studies have found that it is not present in the VTA (Allen Mouse Brain Atlas; Lein et al., 2007) (Ciccarelli et al., 2012; Fritschy & Mohler, 1995; Guyon et al., 1999; Hörtnagl et al., 2013; Okada et al., 2004; Pirker et al., 2000). Although these are the GABA_A_ receptor subunits classically associated with tonic inhibition, it is important to note, however, that there are other examples of neurons that exhibit tonic currents that do not involve these subunits. For instance basolateral nucleus principal cells of the amygdala exhibit α3 GABA_A_ receptor‐mediated tonic currents, and the epileptic hippocampal dentate granule cells were found to maintain tonic currents with α4γ2 subunit containing GABA_A_ receptors (Brickley & Mody, 2012; Marowsky et al., 2012; Rajasekaran et al., 2010). In this case, what is the subunit composition of GABA_A_ receptors mediating tonic inhibition in VTA dopamine neurons? Because we observed some sensitivity to zinc, we hypothesized that GABA_A_ receptors mediating tonic inhibition do not contain γ subunits, consistent with them being located extrasynaptically. One possibility is that they contain only α and β subunits. However, the absence of much effect of THIP and the lack of spontaneous opening suggests that this is not the case (Stórustovu & Ebert, 2006). One interesting possibility is that they contain Ɛ subunits, which are relatively uncommon (e.g., Belujon et al., 2009). Indeed, αβƐ containing receptors exhibit insensitivity to THIP and intermediate sensitivity to Zn^2+^ (Thompson et al., 1998, 2002; Whiting et al., 1997). Our immunostaining results, using an approach optimized for detecting receptor subunit expression in the VTA, revealed extensive expression of Ɛ in the VTA, colocalized with dopamine neurons but not synaptic markers. Furthermore, we found that the α4 subunit in particular was expressed extrasynaptically in dopamine neurons. Therefore, based on our combined pharmacology and immunostaining results, taking into account the existing literature concerning possible subunit combinations and their properties, we propose that the tonic current in VTA dopamine neurons is mediated via extrasynaptic GABA_A_ receptors composed of a relatively unusual α4βƐ subunit combination. This presents a useful working hypothesis that will need to be tested using further pharmacological, anatomical and electrophysiological approaches, ideally combined with subunit‐and cell‐type‐specific genetic deletions.

We also examined the functional consequences of tonic inhibition on dopamine neuron firing. We observed that by increasing tonic inhibition, dopamine neuron excitability was increased. Importantly, we observed these effects when neurons were depolarized from the same resting membrane potential and it is, therefore, not simply a consequence of the hyperpolarizing effect of the tonic current. The most straightforward explanation is that the tonic current increased cell conductance, thereby shunting depolarizing input and making firing less likely. It is tempting to speculate that some of the effects of GABA_A_ receptor blockage on dopamine neuron firing in vivo may involve this tonic current (for example, increased burst firing; Paladini & Tepper, 1999). Moreover, in vivo under conditions of globally increased GABAergic synaptic inputs it may be that this tonic current is increased, thereby reducing excitability and shunting excitatory synaptic inputs. Consistent with this, dopamine neuron input resistant measurements in vivo (Grace & Bunney, 1983) are considerably lower than those taken in vitro (Johnson & North, 1992; Yung et al., 1991). Another possible explanation is that a balance of inhibitory and excitatory tonic currents itself are contributing to pacemaking activity. In dynamic clamp experiments, for instance, either an increase in tonic NMDA or a decrease in tonic GABA currents can produce a burst firing of midbrain dopamine neurons and balanced excitation and inhibition results in pacemaking firing (Canavier & Landry, 2006; Komendantov et al., 2004; Lobb et al., 2010). Furthermore, a high ratio of tonic GABA/NMDA leads to silence and a high ratio of tonic NMDA/GABA leads to bursting. Thus, the level of tonic GABAergic current level may regulate silencing and firing of dopamine neurons and this mechanism could contribute, for example, to pauses in dopamine neuron firing in response to reward omissions and aversive stimuli (Henny et al., 2012; Schultz, 1986; Ungless et al., 2004).

There is emerging evidence that tonic inhibition through GABA_B_ receptors can regulate neuronal firing (Khatri et al., 2019; Wang et al., 2015). Wang et al. reported that GABA_B_ receptor‐mediated tonic inhibition has a direct GABA_A_ receptor‐independent regulatory role on the spontaneous activity of noradrenaline neurons in the locus coeruleus. On the other hand, tonically active GABA_B_ receptors enhance extrasynaptic GABA_A_ receptor‐mediated tonic currents by increasing desensitization and trafficking of GABA_A_ receptors in cerebellar granule cells (Khatri et al., 2019). These findings suggest that GABA_B_ receptors also have a role for tonic inhibition and this can modulate the function of postsynaptic GABA_A_ receptors. Since we demonstrated here that GABA_A_ receptor mediating tonic inhibition in VTA dopamine neurons potentially have an unusual combination of subunits, it would be interesting to see how this rare type of GABA_A_ receptor would interact with GABA_B_ receptor on pacemaking mechanisms of midbrain dopamine neurons.

In conclusion, we reveal the existence of an endogenous tonic GABAergic current in VTA dopamine neurons, which is a result of firing activity in VTA GABA neurons, is potentially mediated by GABA_A_ receptors containing a relatively unusual combination αβƐ subunits, and which can ultimately regulate VTA dopamine neuron firing activity.

## CONFLICTS OF INTEREST

The authors declare no competing financial interests.

## AUTHOR CONTRIBUTIONS

K.T. and M.A.U. designed the research, analysed the data and wrote the paper. K.T. conducted electrophysiological experiments. K.T., R.A.D., B.G. and O.T. conducted anatomical experiments.

### PEER REVIEW

The peer review history for this article is available at https://publons.com/publon/10.1111/ejn.15133.

## Data Availability

All data will be provided on request to corresponding author (kyoko.tossell@imperial.ac.uk).
